# Plasma of Argon Treatment of the Implant Surface, Systematic Review of In Vitro Studies

**DOI:** 10.3390/biom12091219

**Published:** 2022-09-01

**Authors:** Massimo Carossa, Davide Cavagnetto, Francesca Mancini, Alessandro Mosca Balma, Federico Mussano

**Affiliations:** 1 Bone and Dental Bioengineering Lab, Department of Surgical Sciences, CIR Dental School, University of Torino, Via Nizza 230, 10126 Torino, Italy; 2 Politecnico of Torino, Corso Duca degli Abruzzi 24, 10129 Torino, Italy; 3 Private Practice, Via Venezia 7, Pieve Emanuele, 20090 Milan, Italy

**Keywords:** argon, adsorption, biocompatible materials, titanium, osteoblast, fibroblast

## Abstract

This paper aims to review the evidence of the cellular activity on titanium samples exposed to Plasma of Argon (PoA) treatment. A systematic review was carried out based on the PRISMA statement by searching the Cochrane Library, PubMed, Web of Science, EMBASE and Scopus, up to October 2020. Papers were selected according to PICOS format that is: Population (P): osteoblasts, fibroblasts, gingival cells; Intervention (I): PoA disinfection treatment; Comparison (C): untreated controls; Outcome (O): cell culture; Setting (S): in vitro assays. The quality assessment was performed according to the CRIS Guidelines (Checklist for Reporting In vitro Studies). A total of 661 articles were found, of which 16 were included. The quality assessment revealed an overall poor quality of the studies analyzed. In vitro studies on the potential of PoA showed a potential effect in promoting higher cell adhesion and protein adsorption in the earliest times (hours). This outcome was not so evident when later stages of cell growth on the surfaces were tested and compared to the control groups. Only one study was conducted in vivo on a human sample regarding abutment cleaning. No meta-analysis was conducted because of the variety of experimental settings, mixed methods and different cell lines studied. PoA seems to be effective in promoting cell adhesion and protein adsorption. The duration of this effect remains unclear. Further evidence is required to demonstrate the long-term efficacy of the treatment and to support the use of PoA treatment in clinical practice.

## 1. Introduction

Implant rehabilitation is considered a successful treatment for most edentulous patients. Titanium is widely used as a gold standard implant material, which allows predictable clinical outcomes [1]. However, even if the long-term success of this rehabilitation is demonstrated, researchers are continuously focusing on how to improve, from biological to clinical relevance, it’s potential. The long-term success rate of implant rehabilitation is determined by several factors [2,3,4]. Among others, the creation and maintenance of both osseointegration [5] and soft tissue sealing [6,7] around the implant are fundamental. In order to obtain these two key factors, correct and early cell adhesion is mandatory. Surface treatment [8,9,10] of the implant and its abutment have been investigated to obtain a more efficient and stable cell adhesion. Among all the properties that can influence cell response, such as surface topography and chemistry, hydrophilicity certainly covers an important role [11]. The benefits of high surface energy have been highlighted in terms of protein adsorption and early cell adhesion several times [12,13,14]. However, due to the time-dependent interaction with atmospheric hydrocarbon [15], the surface energy tends to decrease, possibly affecting the biological interaction [16,17]. In addition, since the customization process may be more prone to ambient contamination, carbon, aluminum, and siliceous particles, have been found on the titanium surface [18]. These particles are capable to hinder cell response [19] and their presence should be limited, otherwise implant surface may become more hydrophobic [20,21]. Different implant surface treatments, such as ultraviolet light [22,23,24,25,26] and plasma of Argon (PoA) [27], have been studied over the years in order to decontaminate the surface, increase wettability and enhance cell adhesion. In vitro, different researches have shown how PoA is able to activate the surfaces [28] thus increasing cell spreading and affecting the quantity of protein absorbed [29]. Moreover, these effects are achieved without changing the surface topography in order to predispose the best condition for optimal cell adhesion [30]. In vivo studies [31,32] also showed clinical benefits influencing hard and soft tissue levels and cell adhesion. In order to analyze all the results that have been described over the years, in the present study the authors aimed to perform a systematic review of the literature on PoA.

## 2. Materials and Methods

### 2.1. Protocol

This study was performed in accordance with PRISMA-P guidelines for systematic reviews and meta-analyses [33]. 

### 2.2. Information Sources and Search Strategy

The electronic search was conducted under the guidance of a librarian following the available guidelines [34,35]. A structured electronic search of the following electronic databases was performed on 6 October 2020 with specific search strategies (Table 1) for each database: Cochrane Library, PubMed, Web of Science, EMBASE and Scopus.

Hand search among references of included papers, related articles and grey literature was also performed but did not add any new paper or report. No publication date restriction was set and only papers in English were considered. Corrections specific to each database were performed to adapt the query in combination with database-specific filters, where available. 

This review included papers according to the following PICOS format [35] that is: Population (P): osteoblasts, fibroblasts, gingival cells; Intervention (I): PoA disinfection treatment; Comparison(C): untreated controls; Outcome (O): cell culture; Setting (S): in vitro assays. 

### 2.3. Study Selection and Data Collection Process

Papers have been selected according to inclusion criteria developed on the basis of the aforementioned PICOS checklist. Two conditions were set for inclusion: (I) in vitro hard and/or soft tissue periodontal cell culture and (II) comparison between PoA treatment and not-treated control. 

The exclusion criteria were: review articles, case reports, retrospective studies, clinical studies, letters to the editor, personal opinions, guidelines, surveys, conferences and in vivo animal studies. All titles and summaries of studies retrieved in the initial search were evaluated and selected after removing duplicates according to eligibility criteria. The titles and summaries were independently selected by two reviewers (M.C. and Fr. M.), and the papers that met the inclusion criteria were identified and their inclusion was cross-checked by reading the full text to confirm their eligibility. Any disagreement between the two reviewers was solved through discussion. In case of further disagreement, a third reviewer (F.M.) who did not know the opinions of the other two reviewers was contacted and his opinion was determinant to decide whether the paper should be included. For data collection, relevant pieces of information were summarized into a spreadsheet of Microsoft Excel 2016: year of publication and authors, types of cell lines, cell culture methodologies and study outcomes. 

### 2.4. Quality Assessment

To date, there is no standard Risk of Bias tool to assess the methodological quality of in vitro study [36]. The CRIS Guidelines [35] (Checklist for Reporting In vitro Studies) were developed around the necessity of standardized guidelines for improving quality and transparency in reporting and summarizing in vitro dental research. In this review, the CRIS checklist was applied to evaluate the inherent methodological reliability. It consists of five dichotomous questions, which evaluate the methodological aspects of each paper, that is sample size calculation, consideration of the meaning difference required to compute sample size, information on sample loss, clear description of allocation concealment, whether the outcome assessment is performed in blind.

## 3. Results

### 3.1. Search Results

**Figure 1:**
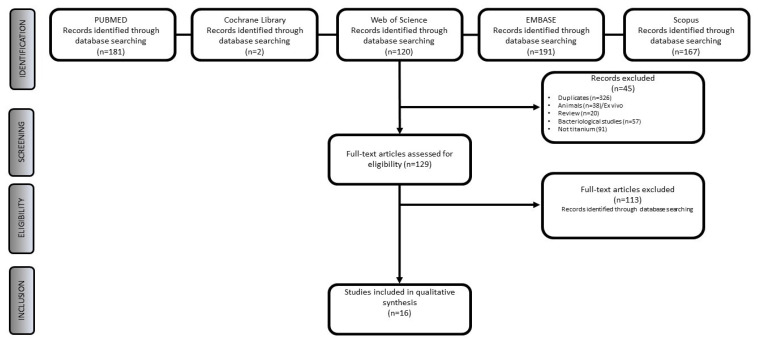
PRISMA flowchart.

A total of 661 titles were found. After removing duplicates (326), title and abstract selection were carried out on 335 papers, of which 129 articles were selected for the evaluation of the full text. A total of 16 papers satisfied the inclusion criteria. The PRISMA flowchart reporting the selection process in detail is reported in Figure 1.

Titles of included studies, studied cell line and CRIS checklist are summarized in Table 2. The main characteristics of included studies are summarized in Table 3.

As there was variation with regard to the methods between various papers as well as differences in the measurement of outcomes, it was impossible to perform any quantitative synthesis of the studies. Therefore, only qualitative descriptive analysis of the aforementioned papers is reported hereafter.

### 3.2. Results of the In Vitro Studies

In vitro studies on the potential of PoA showed a significant effect in promoting higher cell adhesion and protein adsorption in the earliest times (hours). This outcome was not so evident when later stages of cell growth on the surfaces were tested and compared to the control groups. 

Even though no conclusive evidence could be drawn because of heterogeneity issues, it appears that all the studies taken into consideration conclude that PoA treatment is capable to increase the surface free energy of the treated surface. However, the duration of its effects appears to be limited in time.

## 4. Discussion

Implant osseointegration is a complex biological process in which cell spreading and attachment play a paramount role to ensure direct bone apposition to the fixture. In the last decades, several research strategies have focused on the enhancement of this process starting from the bone-implant interface features. Among the most promising technologies available to clinicians, one must take into consideration the chair side use of PoA before implant placement. The assumption of this technique is based simply upon that by increasing the surface energy of a given fixture it is possible to increase its wettability and consequently enhance cell adhesion, as a key preliminary phase leading the osseointegration. Hence, in the present literature review, we searched and identified articles assessing and comparing the biological response of cells seeded onto titanium samples either exposed or not to PoA. Although the heterogeneity of the experimental setting adopted in the papers examined (i.e., different cell types, titanium grades, time of exposure, PoA systems, measured outcomes) may preclude a whole metanalytic approach, a similar effect on cell activity was documented. All the authors reported faster cell adhesion on PoA-treated surfaces. This outcome was similar both in murine and human osteoblasts and fibroblasts, which highlighted the potential benefits of using PoA for the treatment of titanium intrabony fixtures and abutments. 

Pistilli et al. [41] evaluated the effect of PoA treatment on three types of titanium surfaces (machined, double-etched Ti-AE and zirconium nitride) on the adhesion and proliferation of preosteoblastic cells and bacteria. Scanning electron microscopy (SEM) demonstrated a significant augmentation of colony forming units in machined and zirconium nitride surfaces. A sample size calculation was reported, but no detailed information regarding the source of the true difference between the experimental and the control mean was clearly provided. 

As demonstrated by Canullo et al. [29], the increased cell adhesion elicited by PoA is time-dependent, and maximal soon after treatment, decreasing by the first 48 h. This seems not detrimental since the physiologic protein cascade reaches the highest level in the first 48 h [46], and the PoA treatment may be able to exert its action when most required, thus improving the hard and soft tissue healing process. This, however, might indicate also a major susceptibility to bacterial colonization even though it is unlikely to happen immediately after the insertion of the fixture unless gross clinical errors occur and has no influence on second-stage contamination since PoA has an effect only in the short term. No clear indication of the machine used nor the specific protocol used to perform PoA treatment was given in this study. 

González-Blanco et al. [40] assessed the changes occurring in human osteoblasts on Grade IV and V titanium with a sand-blasted, acid-etched (SLA) surface treatment. They found out that cell viability was better in presence of surfaces treated with a vacuum cold PoA (Plasma Finish, PINK GmbH Thermosysteme, Wertheim, Germany) until 48 h when the measured cell viability no longer differed between groups treated with PoA and negative controls. Fluorescence microphotographs used for morphological analysis were performed only at the 24 h incubation period demonstrating a greater average cell area in comparison with surfaces not treated with PoA. The evaluation at the confocal microscope found that cells seeded on the titanium discs with a PoA treatment were enlarged compared to the ones on non-treated surfaces, this event is allegedly due to an improvement in surface hydrophilicity and increased surface energy that favors cell spreading. The different surfaces treated with PoA showed different mitochondrial activity: grade IV titanium treated with PoA showed higher values compared with controls, and grade V titanium showed instead the highest mitochondrial activity in non-treated samples. No clear explanation of this event is given by the authors in the discussion of their paper but stating as a limitation of the study to have a sample reduced in size. Therefore all the considerations taken from this study should be considered with caution. No sample size calculation was reported, thus limiting the internal and external validity of the conclusion of the study, nor was this substantial limitation discussed in the article. The analyzed sample is consistently reduced in size, which hinders the meaningfulness of inferential statistics reported.

Wang et al. [38] stated that the aim of their study was to investigate the capability of low-temperature PoA to maintain the activity of titanium dental implants and to restore the biological functions of implant materials after aging, thus accelerating the biological response of the peri-implant tissues to the implant. 

Canullo et al. [37] aimed to assess whether PoA and ultraviolet light were able to have an impact on cell adhesion (fibroblast) on four different grade 5 titanium surfaces used for the manufacturing of healing abutments. The tested surfaces were machined surfaces and micro-grooved surfaces that had Ultrathin Threaded Microsurface; Anodized Ultrathin Threaded Microsurface or Thin Machined. However, no specification was given regarding the type of titanium surface treatment. These surfaces were divided into three groups: negative control, UV treatment and PoA (8 W and atmospheric pressure for 6 min, using a PoA reactor, Plasma R, Diener Electronic GmbH, Ebhausen, German). The sample size was reported and correctly performed. PoA had an effect on fibroblast morphology in all surfaces only during the first phases of adhesion (showing marked differences after 20 min, but a progressive reduction after 72 h, a time point in which the quantitative differences between treated and control discs were strongly reduced), UV did not produce any significant effect. With regards to cell adhesion, the differences statistically evident after 20 min were no longer present at the other evaluation time points, and no differences between surfaces were noted. 

Henningsen et al. [42] performed a similar comparison between non-thermal plasma (NTP) and UV treatment. This study provided no sample size calculation nor a precise indication of the sample. In vitro cell culture studies are the first approach to test the safety of new methodologies before being approved for further pre-clinical and clinical assessments. The in vivo effects of PoA on titanium implants have been investigated in animal studies. Naujokat [47] analyzed the osseointegration of implants treated with cold PoA in pigs; these results showed slightly higher osseointegration when compared to non-treated implants. This is in an agreement with Canullo [48] and in contrast with Yi-Wen Hung [49] whose study investigated the effect of nonthermal PoA on sandblasted implants in beagle dogs, resulting in non-different implant stability and bone formation compared to the control group. 

In vivo effects of PoA on titanium abutments have also been investigated in humans. Canullo et al. [31] led a randomized control trial over the span of two years to investigate the effect of PoA cleaning on implant abutments. This finding highlighted how the cleaning treatment may be favorable in terms of hard tissue level changes, confirming the results of the first 18 months of the study [17]. From a soft tissue standpoint, Garcia [30] investigated the influence of the PoA cleaning procedure on the soft tissue-abutment interaction in a randomized controlled study. The results indicated positive cell promotion and collagen fiber orientation, in accordance with a previous study by Canullo [32]. In accordance with a recent systematic review by Sanz Martin [50], all the in vivo studies obtained the results using one abutment—a one-time prosthetic approach.

In an in vitro study, Annunziata et al. [51] demonstrated how PoA is also capable of eliminating bacteria contaminations, suggesting its possible role in the treatment of peri-implantitis. This result was also confirmed in the in vivo study by Garcia et al. [30], which observed a more sterilized surface of the group cleaned with the PoA than the control group. This is also in agreement with Canullo et al. [13], who hypothesized the positive effects of PoA in patients with a history of peri-implantitis.

### Limitations of the Study

The inner limitations of the present systematic review are related to the study design of the available studies on the subject, which are mainly in vitro studies and few in vivo studies. In fact, only one study was conducted in vivo on a human sample regarding abutment cleaning. The main question left unanswered by this paper is the net effect of PoA treatment on clinical practice. No meta-analysis was conducted because of the variety of experimental settings, mixed methods and different cell lines studied. Quality assessment of included studies revealed a low score, so a better adherence to shared guidelines in conducting in vivo and in vitro studies is advisable to be able to draw more solid evidence in the future. As a final note, even if sometimes not clearly stated, many authors declaring no conflict of interest appears to have several connections to the manufacturing companies of PoA devices (e.g., the companies producing the device sponsoring their private clinical courses on the same or on related subjects). While other forms of potential bias in biomedical research have been addressed through new processes and policies in the last decade, new approaches to identify and account for potential conflicts of interest are urged to ameliorate the objectivity of scientific literature [52].

## 5. Conclusions

Within the limitations of this systematic review, even though the quality of the most manuscript is in some cases low, most of them state that PoA seems to be a probable method to promote cell adhesion and protein adsorption, especially in the earliest time (hours). 

The duration of this effect remains unclear and further research is needed to demonstrate the long time efficacy of the treatment. However, the results are to be interpreted with caution due to the low percentage of clinical human studies. In accordance, more in vivo studies are required to improve the scientific evidence on this topic and a better quality of in vitro study is advisable.

## Figures and Tables

**Table 1:** Search strategy.

Database	Pubmed	Scopus	Web of Science	Embase	Cochrane Library
Electronic search	(((“plasma”[MeSH Terms] OR “plasma”[All Fields]) OR “plasmas”[All Fields]) OR “plasma s”[All Fields]) AND ((“argon”[MeSH Terms] OR “argon”[All Fields]) OR “argons”[All Fields]) AND “dent*”[All Fields]	TITLE-ABS-KEY (plasma AND argon AND dental)	TOPIC: (plasma AND argon) AND TOPIC: (dental) Timespan: All years. Indexes: SCI-EXPANDED, SSCI, A&HCI, CPCI-S, CPCI-SSH, BKCI-S, BKCI-SSH, ESCI, CCR-EXPANDED, IC.	(‘plasma argon’ OR ((‘plasma’/exp OR plasma) AND (‘argon’/exp OR argon))) AND (‘dental’/exp OR dental)	plasma argon dental
Results	181	167	120	191	2

**Table 2:** Titles of included studies, studied cell line and CRIS checklist.

Title	Object of the Study	D.1 *	D.2 †	D.3 ‡	D.4 §	D.5 ||	Total
Canullo 2020	Human dermal Fibroblast	Y	Y	Y	Y	N	4/5
Wang 2020	Murine Osteoblasts	N	N	N	N	N	0/5
Guo 2019	Murine fibroblast	N	N	N	Y	N	0/5
González-Blanco 2019	Murine Osteoblasts	N	N	N	Y	N	1/5
Pistilli 2018	Murine Osteoblasts	Y	Y	N	Y	N	3/5
Canullo 2017	Murine Osteoblasts/bacteria	Y	Y	Y	Y	N	4/5
Henningsen 2018	Murine Osteoblasts	N	N	N	Y	N	1/5
Canullo 2017	Murine Osteoblasts	Y	Y	N	Y	N	3/5
Garcia 2016	Human soft tissue	N	N	N	Y	Y	2/5
Canullo 2016	Murine Osteoblasts	Y	Y	Y	Y	N	4/5
Canullo 2014	Human soft tissue	N	N	Y	Y	Y	3/5
Canullo 2013	Murine Fibroblastic cells	N	N	Y	N	N	1/5
Duske 2012	Human Osteoblastic cells	N	N	Y	N	N	1/5
Stanford 1994	Murine Osteoblastic cells	N	N	Y	N	N	1/5
Michaels 1991	Murine Periodontal ligament fibroblast	N	N	Y	N	N	1/5
Swart 1992	Murine Osteoblastic cells	N	N	Y	N	N	1/5

* D1: Does the study include a sample size calculation as one of the steps in methodology?, † D2: Is the meaning difference required to compute sample size taken into consideration and discussed?; ‡ D3: Is information on sample loss given? § D4: Is the allocation concealment clearly described?; || D5: Is the outcome assessment performed blind.

**Table 3:** Characteristics of the included studies.

First Author-Year	Study Type	Sample	Plasma System	Time of Exposure	Cell Type	Study Controls	Parameters Assessed	Evaluation Time	Outcome
Canullo 2020 [37]	In vitro	Grade 5 titanium discs with four different topographies (MAC, UTM, UTM-Y, XA)	Plasma reactor (Plasma R, Diener Electronic GmbH, Ebhausen, Germany) at 8 W and atmospheric pressure	6′	Normal Human Dermal Fibroblasts	UV treatment, no treatment	Cell culture, Cell morphology, Adhesion Test, Wettability, SEM	20′, 24 h, 72 h	Data showed potential biological benefits of treating implant abutment surfaces with the PoA in relation to early-stage cell adhesion.
Wang 2020 [38]	In vitro	Titanium grade 4 titanium disk	Atmospheric Pressure Plasma System model AS400 + PFW10, manufactured by Plasma Treat GmbH (Steinhagen, Germany)	90″	Osteoblast rat cells	No treatment	Surface Morphology, Surface Hydrophilicity, Surface Chemistry, Adhesion and Spreading, OCN Assay	12 h for the osteoblast morphology, 24 h for actin (spreading behavior and cytoskeletal arrangement), 7 and 14 days for OCN	Osteoblast cells’ adhesion, proliferation, and mineralization were all significantly improved. The low-temperature PoA treatment could be a potentially effective approach to activate titanium-based dental implants for improved performance.
Guo 2019 [39]	In vitro	Titanium grade 4 discs, tetragonal zirconia polycrystal discs and PEEK discs	NTP reactor (generator frequency 100 kHz, input power 24 W, system pressure 1 mbar, gas flow rate 1.25 sccm, and gas purity >99.5% Diener Electronic GmbH, Ebhausen, Germany).	12′	L929 murine fibroblast cells and human gingiva fibroblast cells	UV treatment, no treatment	Cell culture, Cell Attachment and Morphology, Viability Assay, Cytotoxicity Assay	2 h, 24 h, 48 h	Oxygen plasma treatment may improve the attachment, proliferation and viability of soft tissue cells. PoA treatment showed only minor effects on the cytocompatibility of soft tissue cells.
González-Blanco 2019 [40]	In vitro	Grade 4 and 5 SLA titanium discs	V15-G plasma reactor produced by Plasma Finish (PINK GmbH Thermosysteme, Wertheim, Germany), placed into an ISO 7 clean room (Lesatec, Opera Milan, Italy), pressure 20 Pa.	15′	Osteoblast MG-63 cell line	No treatment	Cell Culture, Cell Viability Analysis, Morphological Analysis and Mitochondrial Energy Balance	6 h, 24 h, 48 h	The use of argon plasma as an intervention for decontaminating the surfaces of titanium implants may lead to an improvement in the growth, cell size, spreading and mitochondrial activity of the MG-63 cells that cover them.
Pistilli 2018 [41]	In vitro	Grade 4 titanium discs	Plasma reactor (Plasma R, Sweden & Martina), 10 W, 1 bar	12′	Murine preosteoblasts (MC3T3-E1)	No treatment	SEM, Surface roughness analysis, Cell Adhesion (CA), Protein Adsorption (PA), Bacterial Biofilm Evaluation (BE)	20 min for CA, 30 min for PA, 24 h for BE)	PoA treatment significantly increased the protein adsorption level. Rough implant surfaces benefited the most from the PoA treatment.
Canullo 2017 [14]	In vitro	Grade 4 titanium discs with different surface modifications (MAC, TPS and ZRT)	APDBD treatment (8 W at atmospheric pressure) using a nonthermal dielectric barrier discharge (Plasma Beam Mini, Diener Electronic).	2′	Preosteoblastic murine cell line MC3T3-E1	No treatment	Contact angle, Bacterial Adhesion, Protein Adsorption (PA), Cell culture, Cell Adhesion Assay (CA), Cell Morphology (CM), Viability Assay (VA)	30′ for PA; 12′ for CA; 30′ and 8 h for CM; 24 h, 48 h, 72 h for VA	Argon atmospheric pressure dielectric barrier discharge showed the ability to enhance osteoblast attachment and spreading as well as bacterial decontamination.
Henningsen 2018 [42]	In vitro	Sandblasted and acid-etched grade 4 titanium discs	Yocto III NTP plasma reactor (Diener Electronic GmbH, Ebhausen, Germany). 24 W -0.5 mbar	12′	Murine osteoblast-like cells MC3T3-E1	UV treatment, no treatment	SEM, Surface Roughness Measurements, Wettability, Cell Culture, Cell Attachment and Morphology (CAM), Cell Proliferation (CP), Cytotoxicity, XPS Analysis, Viability	2 h, 24 h, 72 h for CAM; 52 h for Viability; 24 h, 48 h, 72 h for CP and Cytotoxicity	NTP and UV treatments result in an optimized cell environment on titanium disks compared to the non-treated control without conducting any topographical or roughness changes under laboratory conditions.
Canullo 2017 [43]	In vitro	Grade 4 titanium discs with different surface modifications (MAC, TPS and ZRT)	Plasma reactor (Plasma R, Sweden & Martina), 10 W, 1 bar	12′	Preosteoblastic murine cell line MC3T3-E1	No treatment	FESEM, Contact Angle, Cell Culture, Cell Morphology	2 h, 8 h, 24 h for cell morphology	Morphologic changes in adherent osteoblasts could be detected, supporting the efficacy of cold PoA treatment, for all the implant surfaces evaluated.
Garcia 2017 [30]	In vivo	Titanium abutment, 30 patients	Plasma reactor (Diener Electronic GmbH, Jettingen, Germany), 75 W, −10 Mpa	12′	Fibroblast	No treatment	Abutment Surface Analysis, Histological Analysis	2 weeks after a second surgery	PoA may promote cell adhesion and positively influence collagen fiber orientation.
Canullo 2016 [28]	In vitro	Grade 4 titanium disks with different surface modifications (MAC, TPS and ZRT)	Plasma reactor (Plasma R; Sweden & Martina), 10 W, 1 bar	12′	Preosteoblastic murine cell line MC3T3-E1 and human osteoblastic cell line MG-63	UV treatment, no treatment	Topography and Surface Analyses, Protein Adsorption (PA), Cell culture, Cell Adhesion assay (CA)	30′ for PA, 15′ for CA	The present study highlights the potential benefits of treating implant surfaces with PoA (12 min) or UV (3 h)
Canullo 2014 [32]	In vivo	Titanium abutments, 18 patients	Plasma reactor (Diener Electronic GmbH, Jettingen, Germany), 75 W, −10 Mpa	12′	Fibroblast	No treatment, laboratory customization and cleaning by steam	Percentage of the Total Area Occupied by Cells, Presence or Absence of Cells, Aspect of the Adhered Cells and the Presence of Contaminants	One week after a second surgery	Results suggest a better adhesion of soft tissue cells to titanium abutments cleaned by PoA than to those inserted as they come from the industry or cleaned by steam after laboratory customization.
Canullo 2013 [29]	In vitro	Machined grade 5 titanium disks	Plasma reactor (Colibri, Gambetti Company), 10 W, 1 bar	6′	Murine fibroblastic cells (L929)	No treatment	Cell Adhesion, Process of Adhesion and Colonization of the Surfaces	2 h, 8 h, 48 h	PoA treatment on titanium disks immediately before exposure to a suspension of L929 murine fibroblastic cells significantly increased the speed of cellular adhesion compared to untreated control disks.
Duske 2012 [23]	In vitro	Grade IV Titanium disks with different topographies	Atmospheric pressure plasma jet (INP Greifswald, Greifswald, Germany), with a frequency of applied voltage of 1.82 MHz with an input power of 2–3 W	30″, 60″, 120″	Human osteoblastic cells (MG-63, ATCC, CRL-1427)	Machined (M), sandblast-etched (SLA) and discs with a hydrophilic SLActive^®^ surface (ACT)	Contact Angle Measurement, Cell Culture, Spreading, Metabolic Activity and SEM of Human Osteoblastic Cells (MG-63)	30′, 60′ and 24 h	Results suggest that a PoA with a small oxygen admixture was effective for surface modifications resulting in favourable cell responses.
Stanford 1994 [44]	In vitro	Commercially pure Titanium samples	Plasma discharge chamber (model PDC-32G Plasma Cleaner, Harrick Scientific Corporation, Ossining, NY), 100 W, 0.07 Mpa	5′	Rat calvarial osteoblast-like cells	UV light, autoclave, ethylene oxide gas	Cell culture, Osteocalcin RIA, Collagen Expression, Alkaline Phosphatase Enzyme Activity, Alizarin Red Calcium Assay	4, 6, 8, 10, 12 days	Osteocalcin and alkaline phosphatase, but not collagen expression, were significantly affected by surface roughness when these surfaces were altered by PoA-cleaning. In general, PoA-cleaned cpTi surfaces demonstrated an inverse relationship between surface roughness and phenotypic markers for a bone-like response
Michaels 1991 [45]	In vitro	Commercially pure Titanium disks	Harrick plasma-cleaning device (Harrick Scientific, Ossininf, NY 10562, USA)	1′, 5′	Rat periodontal ligament fibroblast-like cells	UV light, autoclave, ethylene oxide gas	Cell Attachment Assay, SEM Evaluation of Cellular Spreading	15′, 30′, 60′, 120′	PoA-cleaning for up to five min did not appear to enhance cell attachment, but it benefited spreading.
Swart 1992 [27]	In vitro	Commercially pure Titanium disks	Harrick PDC-32G plasma cleaning device (Harrick Scientific Corp., Ossining, NY, USA), Ar 100 W, 0.15 atm	1′, 5′, 10′	Osteoblast like cells	No treatment	Cell Attachment Assays, SEM, X-ray Photoelectron Spectroscopy, Auger Electron Spectroscopy, Contact Angle	15′, 30′, 60′, 120′	Short-term PoA-cleaning treatments may produce a relatively contaminant-free, highly wettable surface that favors early in vitro osteoblast-like cellular attachment and morphological integration.

## Data Availability

Data are available upon request from the first author email address after reasonable request.

## References

[1] Lindquist L.W., Carlsson G.E., Jemt T. (1996). A prospective 15-year follow-up study of mandibular fixed prostheses supported by osseointegrated implants. Clinical results and marginal bone loss. Clin. Oral Implant. Res..

[2] Jimbo R., Albrektsson T. (2015). Long-term clinical success of minimally and moderately rough oral implants: A review of 71 studies with 5 years or more of follow-up. Implant Dent..

[3] Madani E., Smeets R., Freiwald E., Sanj M.S., Jung O., Grubeanu D., Hanken H., Henningsen A. (2018). Impact of different placement depths on the crestal bone level of immediate versus delayed placed platform-switched implants. J. Cranio-Maxillo-Facial Surg. Off. Publ. Eur. Assoc. Cranio-Maxillo-Facial Surg..

[4] Gherlone E.F., D’Orto B., Nagni M., Capparè P., Vinci R. (2022). Tilted Implants and Sinus Floor Elevation Techniques Compared in Posterior Edentulous Maxilla: A Retrospective Clinical Study over Four Years of Follow-Up. Appl. Sci..

[5] Albrektsson T., Brånemark P.I., Hansson H.A., Lindström J. (1981). Osseointegrated titanium implants. Requirements for ensuring a long-lasting, direct bone-to-implant anchorage in man. Acta Orthop. Scand..

[6] Suárez-López Del Amo F., Lin G.-H., Monje A., Galindo-Moreno P., Wang H.-L. (2016). Influence of Soft Tissue Thickness on Peri-Implant Marginal Bone Loss: A Systematic Review and Meta-Analysis. J. Periodontol..

[7] Welander M., Abrahamsson I., Berglundh T. (2008). The mucosal barrier at implant abutments of different materials. Clin. Oral Implant. Res..

[8] Rompen E., Domken O., Degidi M., Pontes A.E.F., Piattelli A. (2006). The effect of material characteristics, of surface topography and of implant components and connections on soft tissue integration: A literature review. Clin. Oral Implant. Res..

[9] Wennerberg A., Albrektsson T. (2009). Effects of titanium surface topography on bone integration: A systematic review. Clin. Oral Implant. Res..

[10] Kang B.-S., Sul Y.-T., Oh S.-J., Lee H.-J., Albrektsson T. (2009). XPS, AES and SEM analysis of recent dental implants. Acta Biomater..

[11] Eriksson C., Nygren H., Ohlson K. (2004). Implantation of hydrophilic and hydrophobic titanium discs in rat tibia: Cellular reactions on the surfaces during the first 3 weeks in bone. Biomaterials.

[12] Henningsen A., Smeets R., Heuberger R., Jung O.T., Hanken H., Heiland M., Cacaci C., Precht C. (2018). Changes in surface characteristics of titanium and zirconia after surface treatment with ultraviolet light or non-thermal plasma. Eur. J. Oral Sci..

[13] Canullo L., Genova T., Wang H.-L., Carossa S., Mussano F. (2017). Plasma of Argon Increases Cell Attachment and Bacterial Decontamination on Different Implant Surfaces. Int. J. Oral Maxillofac. Implant..

[14] Lee J.H., Ogawa T. (2012). The biological aging of titanium implants. Implant Dent..

[15] Textor M., Sittig C., Frauchiger V., Tosatti S., Brunette D.M., Brunette D.M., Tengvall P., Textor M., Thomsen P. (2001). Properties and Biological Significance of Natural Oxide Films on Titanium and Its Alloys. Titanium in Medicine: Material Science, Surface Science, Engineering, Biological Responses and Medical Applications.

[16] Bumgardner J.D., Wiser R., Elder S.H., Jouett R., Yang Y., Ong J.L. (2003). Contact angle, protein adsorption and osteoblast precursor cell attachment to chitosan coatings bonded to titanium. J. Biomater. Sci. Polym. Ed..

[17] Canullo L., Götz W. (2012). Peri-implant hard tissue response to glow-discharged abutments: Prospective study. Preliminary radiological results. Ann. Anat..

[18] Kasemo B., Lausmaa J. (1988). Biomaterial and implant surfaces: On the role of cleanliness, contamination, and preparation procedures. J. Biomed. Mater. Res..

[19] Sabetrasekh R., Tiainen H., Reseland J.E., Will J., Ellingsen J.E., Lyngstadaas S.P., Haugen H.J. (2010). Impact of trace elements on biocompatibility of titanium scaffolds. Biomed. Mater..

[20] Hallab N.J., Bundy K.J., O’Connor K., Moses R.L., Jacobs J.J. (2001). Evaluation of metallic and polymeric biomaterial surface energy and surface roughness characteristics for directed cell adhesion. Tissue Eng..

[21] Lampin M., Warocquier-Clérout, Legris C., Degrange M., Sigot-Luizard M.F. (1997). Correlation between substratum roughness and wettability, cell adhesion, and cell migration. J. Biomed. Mater. Res..

[22] Aita H., Hori N., Takeuchi M., Suzuki T., Yamada M., Anpo M., Ogawa T. (2009). The effect of ultraviolet functionalization of titanium on integration with bone. Biomaterials.

[23] Duske K., Koban I., Kindel E., Schröder K., Nebe B., Holtfreter B., Jablonowski L., Weltmann K.D., Kocher T. (2012). Atmospheric plasma enhances wettability and cell spreading on dental implant metals. J. Clin. Periodontol..

[24] Hori N., Ueno T., Suzuki T., Yamada M., Att W., Okada S., Ohno A., Aita H., Kimoto K., Ogawa T. (2010). Ultraviolet light treatment for the restoration of age-related degradation of titanium bioactivity. Int. J. Oral Maxillofac. Implant..

[25] Suzuki T., Hori N., Att W., Kubo K., Iwasa F., Ueno T., Maeda H., Ogawa T. (2009). Ultraviolet treatment overcomes time-related degrading bioactivity of titanium. Tissue Eng. Part A.

[26] Gao Y., Liu Y., Zhou L., Guo Z., Rong M., Liu X., Lai C., Ding X. (2013). The effects of different wavelength UV photofunctionalization on micro-arc oxidized titanium. PLoS ONE.

[27] Swart K.M., Keller J.C., Wightman J.P., Draughn R.A., Stanford C.M., Michaels C.M. (1992). Short-term plasma-cleaning treatments enhance in vitro osteoblast attachment to titanium. J. Oral Implantol..

[28] Canullo L., Genova T., Tallarico M., Gautier G., Mussano F., Botticelli D. (2016). Plasma of Argon Affects the Earliest Biological Response of Different Implant Surfaces: An In Vitro Comparative Study. J. Dent. Res..

[29] Canullo L., Cassinelli C., Götz W., Tarnow D. (2013). Plasma of argon accelerates murine fibroblast adhesion in early stages of titanium disk colonization. Int. J. Oral Maxillofac. Implant..

[30] Garcia B., Camacho F., Peñarrocha D., Tallarico M., Perez S., Canullo L. (2017). Influence of plasma cleaning procedure on the interaction between soft tissue and abutments: A randomized controlled histologic study. Clin. Oral Implant. Res..

[31] Canullo L., Peñarrocha D., Clementini M., Iannello G., Micarelli C. (2015). Impact of plasma of argon cleaning treatment on implant abutments in patients with a history of periodontal disease and thin biotype: Radiographic results at 24-month follow-up of a RCT. Clin. Oral Implant. Res..

[32] Canullo L., Penarrocha-Oltra D., Marchionni S., Bagán L., Peñarrocha-Diago M.-A., Micarelli C. (2014). Soft tissue cell adhesion to titanium abutments after different cleaning procedures: Preliminary results of a randomized clinical trial. Med. Oral Patol. Oral Cir. Bucal.

[33] Shamseer L., Moher D., Clarke M., Ghersi D., Liberati A., Petticrew M., Shekelle P., Stewart L.A. (2015). Preferred reporting items for systematic review and meta-analysis protocols (PRISMA-P) 2015: Elaboration and explanation. BMJ.

[34] Maia L.C., Antonio A.G. (2012). Systematic reviews in dental research. A guideline. J. Clin. Pediatr. Dent..

[35] Krithikadatta J., Gopikrishna V., Datta M. (2014). CRIS Guidelines (Checklist for Reporting In-vitro Studies): A concept note on the need for standardized guidelines for improving quality and transparency in reporting in-vitro studies in experimental dental research. J. Conserv. Dent..

[36] Tran L., Tam D.N.H., Elshafay A., Dang T., Hirayama K., Huy N.T. (2021). Quality assessment tools used in systematic reviews of in vitro studies: A systematic review. BMC Med. Res. Methodol..

[37] Canullo L., Genova T., Gross Trujillo E., Pradies G., Petrillo S., Muzzi M., Carossa S., Mussano F. (2020). Fibroblast interaction with different abutment surfaces: In vitro study. Int. J. Mol. Sci..

[38] Wang L., Wang W., Zhao H., Liu Y., Liu J., Bai N. (2020). Bioactive Effects of Low-Temperature Argon-Oxygen Plasma on a Titanium Implant Surface. ACS Omega.

[39] Guo L., Smeets R., Kluwe L., Hartjen P., Barbeck M., Cacaci C., Gosau M., Henningsen A. (2019). Cytocompatibility of Titanium, Zirconia and Modified PEEK after Surface Treatment Using UV Light or Non-Thermal Plasma. Int. J. Mol. Sci..

[40] González-Blanco C., Rizo-Gorrita M., Luna-Oliva I., Serrera-Figallo M.-Á., Torres-Lagares D., Gutiérrez-Pérez J.-L. (2019). Human Osteoblast Cell Behaviour on Titanium Discs Treated with Argon Plasma. Materials.

[41] Pistilli R., Genova T., Canullo L., Faga M.G., Terlizzi M.E., Gribaudo G., Mussano F. (2018). Effect of Bioactivation on Traditional Surfaces and Zirconium Nitride: Adhesion and Proliferation of Preosteoblastic Cells and Bacteria. Int. J. Oral Maxillofac. Implant..

[42] Henningsen A., Smeets R., Hartjen P., Heinrich O., Heuberger R., Heiland M., Precht C., Cacaci C. (2018). Photofunctionalization and non-thermal plasma activation of titanium surfaces. Clin. Oral Investig..

[43] Canullo L., Genova T., Mandracci P., Mussano F., Abundo R., Fiorellini J.P. (2017). Morphometric Changes Induced by Cold Argon Plasma Treatment on Osteoblasts Grown on Different Dental Implant Surfaces. Int. J. Periodont. Restor. Dent..

[44] Stanford C.M., Keller J.C., Solursh M. (1994). Bone cell expression on titanium surfaces is altered by sterilization treatments. J. Dent. Res..

[45] Michaels C.M., Keller J.C., Stanford C.M. (1991). In vitro periodontal ligament fibroblast attachment to plasma-cleaned titanium surfaces. J. Oral Implantol..

[46] Susin C., Fiorini T., Lee J., De Stefano J.A., Dickinson D.P., Wikesjö U.M.E. (2015). Wound healing following surgical and regenerative periodontal therapy. Periodontol. 2000.

[47] Naujokat H., Harder S., Schulz L.Y., Wiltfang J., Flörke C., Açil Y. (2019). Surface conditioning with cold argon plasma and its effect on the osseointegration of dental implants in miniature pigs. J. Cranio-Maxillo-Facial Surg..

[48] Canullo L., Tallarico M., Botticelli D., Alccayhuaman K.A.A., Martins Neto E.C., Xavier S.P. (2018). Hard and soft tissue changes around implants activated using plasma of argon: A histomorphometric study in dog. Clin. Oral Implant. Res..

[49] Hung Y.-W., Chen H.-L., Lee L.-T., Tung K.-C., Bau D.-T., Wong Y.-K. (2018). Effects of non-thermal plasma on sandblasted titanium dental implants in beagle dogs. J. Chin. Med. Assoc..

[50] Sanz-Martín I., Sanz-Sánchez I., Carrillo de Albornoz A., Figuero E., Sanz M. (2018). Effects of modified abutment characteristics on peri-implant soft tissue health: A systematic review and meta-analysis. Clin. Oral Implant. Res..

[51] Annunziata M., Canullo L., Donnarumma G., Caputo P., Nastri L., Guida L. (2016). Bacterial inactivation/sterilization by argon plasma treatment on contaminated titanium implant surfaces: In vitro study. Med. Oral Patol. Oral Cir. Bucal.

[52] Dunn A.G., Coiera E., Mandl K.D., Bourgeois F.T. (2016). Conflict of interest disclosure in biomedical research: A review of current practices, biases, and the role of public registries in improving transparency. Res. Integr. Peer Rev..

